# Idiopathic Adult Intussusception

**DOI:** 10.4103/1319-3767.70625

**Published:** 2010-10

**Authors:** Pankaj K. Garg

**Affiliations:** Department of Surgery, University College of Medical Sciences and Guru Teg Bahadur Hospital, University of Delhi, Delhi-110 095, India. E-mail: dr.pankajgarg@gmail.com

Sir,

I read with great interest the image quiz “Unusal cause of crampy abdominal pain” by Yagnik.[1] In the answer published in July-September 2010 issue of Saudi Journal of Gastroenterology. In the answer of second question, he mentioned that intussusception in adults is almost always due to pre-existing disease. I like to state here that 8-20% of cases of intussusceptions in adults are idiopathic.[2] In a recent retrospective review of 41 patients of adult intussusceptions by Wang *et al*.,[3] four (9.1%) cases were idiopathic where etiologies could not be found by surgical exploration and/or pathology. These idiopathic intussusceptions in adults usually involve small intestine.
